# Plant Insecticide L-Canavanine Repels *Drosophila* via the Insect Orphan GPCR DmX

**DOI:** 10.1371/journal.pbio.1000147

**Published:** 2009-06-30

**Authors:** Christian Mitri, Laurent Soustelle, Bérénice Framery, Joël Bockaert, Marie-Laure Parmentier, Yves Grau

**Affiliations:** Institut de Génomique Fonctionnelle, CNRS UMR5203, INSERM U661, University of Montpellier I and II, Montpellier, France; Baylor College of Medicine, United States of America

## Abstract

For all animals, the taste sense is crucial to detect and avoid ingesting toxic molecules. Many toxins are synthesized by plants as a defense mechanism against insect predation. One example of such a natural toxic molecule is l-canavanine, a nonprotein amino acid found in the seeds of many legumes. Whether and how insects are informed that some plants contain l-canavanine remains to be elucidated. In insects, the taste sense relies on gustatory receptors forming the gustatory receptor (Gr) family. Gr proteins display highly divergent sequences, suggesting that they could cover the entire range of tastants. However, one cannot exclude the possibility of evolutionarily independent taste receptors. Here, we show that l-canavanine is not only toxic, but is also a repellent for *Drosophila*. Using a pharmacogenetic approach, we find that flies sense food containing this poison by the DmX receptor. DmXR is an insect orphan G-protein–coupled receptor that has partially diverged in its ligand binding pocket from the metabotropic glutamate receptor family. Blockade of DmXR function with an antagonist lowers the repulsive effect of l-canavanine. In addition, disruption of the DmXR encoding gene, called *mangetout* (*mtt*), suppresses the l-canavanine repellent effect. To avoid the ingestion of l-canavanine, DmXR expression is required in bitter-sensitive gustatory receptor neurons, where it triggers the premature retraction of the proboscis, thus leading to the end of food searching. These findings show that the DmX receptor, which does not belong to the Gr family, fulfills a gustatory function necessary to avoid eating a natural toxin.

## Abstract 2

An orphan G-protein-coupled gustatory receptor mediates detection of the plant poison L-canavanine in fruit flies.

## Author Summary

Plants evolve to fend off the insects that attack them, often by synthesizing compounds toxic to insects. In turn, insects develop strategies to avoid these plants or resist their toxins. Some plant toxins are nonprotein amino acids. For example, seeds from numerous legumes contain high amounts of l-canavanine, a nonprotein amino acid that is structurally related to l-arginine and is highly toxic to most insects. How insects can detect l-canavanine remains to be elucidated. Using pharmacology, genetics, and behavioral approaches, we show that flies sense l-canavanine using the receptor DmX, an orphan G-protein–coupled receptor that has diverged in its ligand binding pocket from metabotropic glutamate receptors. Disruption of the DmXR gene, called *mangetout* (*mtt*), suppresses the l-canavanine repellent effect. DmXR is expressed and required in aversive gustatory receptor neurons, where it triggers the premature retraction of the proboscis, thus leading to the end of food searching. Our results indicate a mechanism by which some insects may detect and avoid a plant toxin.

## Introduction

Taste is essential to distinguish between nutritious and toxic substances. To avoid eating toxins, animals are able to detect them by using a repertoire of taste receptors [1]. Although it is recognized that a bitter taste sensation is critical to avoid toxic substances [2],[3], the cellular and molecular mechanisms that have been established during evolution to detect a toxin are not well understood. In particular, how a receptor becomes tuned to a toxin is not well documented, mainly because the structure of its ligand binding pocket (LBP) and the evolutionary relationship with the ancestor receptor are not known.

In *Drosophila*, the family of gustatory receptors (Grs) is predicted to consist of 68 genes [4],[5]. This family of receptors, which consist of seven transmembrane domain proteins, is characterized by a very high level of amino acid divergence, showing as little as 8%–12% amino acid identity [5]. Such diversity suggests that the Gr family could cover the entire range of taste-receptive capability of the fly. Nevertheless, the extreme divergence within this family does not exclude the possibility of evolutionarily independent insect taste receptors not belonging to the Gr family. To date, only few receptors of the Gr family have been associated with a specific taste molecule: for example, the receptor for the sugar trehalose, called Gr5a [6], and the bitter compound caffeine coreceptors, called Gr66a and Gr93a [7],[8].

Plants synthesize many toxic molecules as defense mechanisms against predation [9],[10]. A number of such toxic compounds are nonprotein amino acids [11],[12]. The best-characterized example of nonprotein amino acid that plays a defensive role is l-canavanine (2-amino-4-guanidinooxybutyric acid) [13]–[15], which is massively accumulated in the seeds of many legumes (up to 143 mM in *Medicago sativa* [16]). l-Canavanine is a natural insecticide because it is structurally similar enough to l-arginine (Figure S1) to interfere with l-arginine metabolism and to be incorporated by arginyl-tRNA synthase in de novo proteins resulting in dysfunctional proteins [17]–[19]. Thus, these properties of l-canavanine render it a highly toxic secondary plant constituent [15]. To deal with this natural poison, some insects have generated several adaptive strategies. Indeed, the tobacco budworm *Heliothis virescens* uses detoxification [20] and the beetle *Caryedes brasiliensis* feeds exclusively on l-canavanine–containing seeds but catabolizes l-canavanine to l-canaline and urea [21]. However, these two mechanisms to circumvent the toxic properties of l-canavanine are specific to few insect species. Thus, the evidence for a protective function against predation for such nonprotein amino acids, i.e., whether and how insects are informed that plants contain l-canavanine, remains to be shown [15].

Amino acids are known to be the ligands of G-protein–coupled receptors (GPCRs) belonging to the family C [22],[23]. All members of this family display a common structural architecture characterized by a long N-terminal extracellular domain containing a bilobular LBP [24], a seven transmembrane domain, and an intracellular C-terminus. This family includes metabotropic glutamate receptors (mGluRs). In mammals and in insects, mGluRs, which are activated by the neurotransmitter glutamate, play different roles in the central nervous system [25],[26]. We have previously shown that one mGluR has diverged through evolution to give rise to the mX receptor, called DmXR in *Drosophila* [27]. Orthologs of DmXR are so far only found in insects [27]. DmXR differs from mGluRs in the distal part of the LBP, so that this receptor is an orphan receptor, which is not activated by glutamate [27]. However, we previously showed that the DmXR and mGluR LBPs share the crucial residues necessary to bind a ligand with amino acid structural properties [27]. To deorphanize the DmX receptor, we previously tested various molecules having such properties, including all the classical amino acids, and did not find any ligand [27].

Drosophilidae are saprophytic animals, and members of dipteran families such as Tephritidae or Scatophagidae are seed predators [28], so we asked whether l-canavanine could activate DmXR. Here, we show that l-canavanine is a ligand of DmXR in vitro. We then wondered whether insects could be informed that plants contain l-canavanine via the mX receptor. We have addressed this question by using *Drosophila* as an insect model. First, we confirmed that l-canavanine is highly toxic when ingested. We then tested whether *Drosophila* avoid eating food containing l-canavanine. We found that l-canavanine is recognized by flies and mediates a behavioral avoidance response via a chemosensory mechanism. Hence, l-canavanine is a repellent. We then analyzed the molecular and cellular bases of l-canavanine–induced repulsive behavior, using gustatory behavior, pharmacology, and genetic approaches. We found that l-canavanine is detected in vivo by the DmX receptor. To control the l-canavanine avoidance behavior, the DmX receptor is expressed and required in bitter-sensitive gustatory receptor neurons (GRNs). These findings show that the gustatory detection of a natural toxin relies on DmXR, a divergent mGluR not belonging to the Gr family.

## Results

### The Orphan Insect Receptor DmX Is Activated In Vitro by L-Canavanine

**Figure 1:**
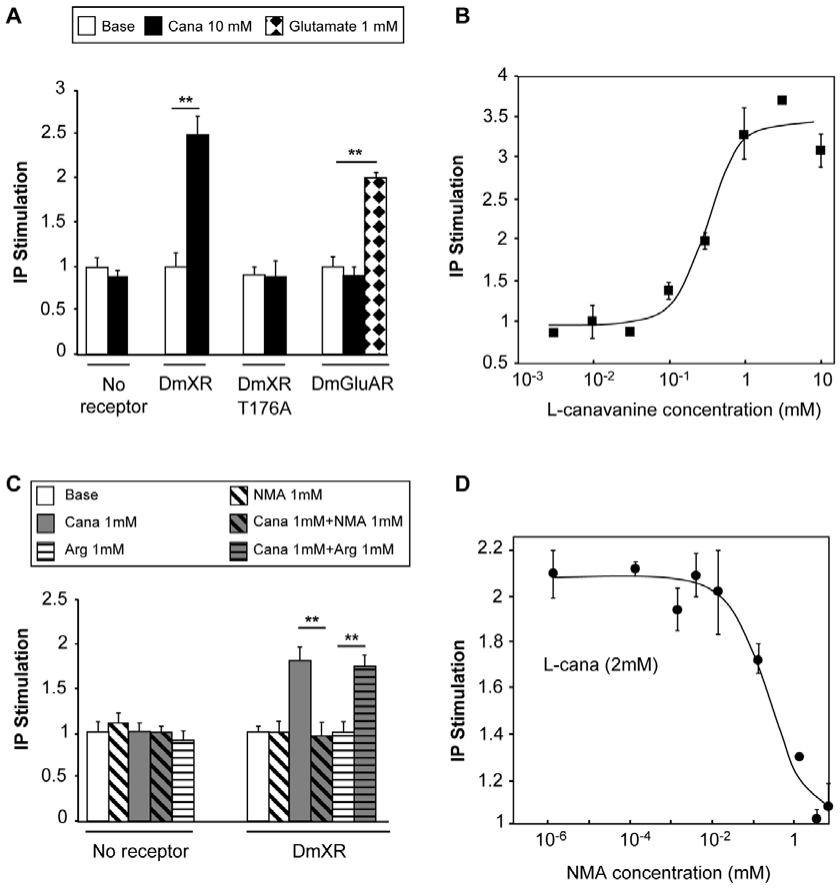
L-Canavanine activates DmXR and *N*-methyl-l-arginine (NMA) inhibits DmXR. (A) l-Canavanine (Cana) is an agonist of DmXR. l-Canavanine at a concentration of 10 mM (black) activated DmXR in HEK cells, but had no effect on the unique fly metabotropic glutamate receptor DmGluA, which was activated by 1 mM glutamate (checkered). Note that 10 mM l-canavanine did not activate the DmX ligand binding pocket mutant receptor (DmXRT176A). (B) Dose-response curve of l-canavanine on DmXR. IP production by DmX receptors activated with increasing concentrations of l-canavanine (half-maximal effective concentration [EC_50_] of l-canavanine = 0.5±0.2 mM). (C) *N*-methyl-l-arginine (NMA) inhibits DmXR. The activation of DmXR by 1 mM l-canavanine (grey) was completely inhibited by 1 mM NMA (diagonally hatched on grey background). Thus NMA, which has no effect by itself (diagonally hatched on white background), is a potent antagonist of DmXR. l-arginine (1 mM) did not activate DmXR (horizontally hatched on white background) nor antagonize 1 mM l-canavanine effect on DmXR (horizontally hatched on grey background). (D) Dose-response curve of NMA antagonistic effect on DmXR. IP production by DmX receptors activated with 2 mM l-canavanine (l-cana) in the presence of increasing concentrations of the antagonist NMA (half-maximal inhibitory concentration [IC_50_] of NMA = 0.2±0.2 mM). For (A–D), data are expressed as the IP (inositol triphosphate) production in HEK cells coexpressing a chimeric G-protein α-subunit Gqi9 and the indicated receptor in presence of drugs relatively to IP production in the basal conditions. The vertical bars represent the standard error of the mean (SEM of triplicate determinations from typical experiments). Asterisks indicate significant differences by *t*-test (*p*<0.001).

To test whether l-canavanine could activate DmXR, we transiently expressed this receptor in human embryonic kidney (HEK) cells and assayed for l-canavanine–induced DmXR activation. We found that l-canavanine activated HEK cells expressing DmXR (Figure 1A and Figure 1B). In contrast, the close structural homolog l-arginine showed no agonist or antagonist effect on DmXR (Figure 1C). l-Canavanine did not activate HEK cells expressing the unique fly mGlu receptor, DmGluA (Figure 1A). We searched for an antagonist and found that *N*-methyl-l-arginine (NMA) inhibited DmXR activation by l-canavanine (Figure 1C and 1D). Previous sequence analysis, mutagenesis, and 3-D modeling studies had shown that the LBP of the DmXR is very homologous to the LBP of mGluRs [27]. The residues contacting the amino acid moiety of glutamate (the α-COO^−^ and NH3^+^ groups) are conserved in DmXR (e.g., Thr-176), whereas the residues interacting with the γ-carboxylic group are not [27]. The Thr-176 residue is conserved in all mGluRs, and its mutation strongly decreases the affinity of these receptors for glutamate [24]. Similarly, l-canavanine did not activate Thr-176–mutated DmX receptor (DmXRT176A) transfected in HEK cells (Figure 1A), although this mutated receptor is actually localized at the plasma membrane (as shown in [27]). This indicates that the plant amino acid binds into the LBP. Altogether our data show that DmXR is a l-canavanine receptor because of a partial modification of its LBP from the LBP of mGluRs. These results suggest that *Drosophila* may be able to detect l-canavanine in vivo through the DmX receptor.

### L-Canavanine Is Toxic for *Drosophila*

Since l-canavanine is described as a natural insecticide [15], we first examined whether ingested l-canavanine is also toxic for *Drosophila melanogaster*. We maintained 50 young wild-type (WT) flies on *Drosophila* medium containing 10 mM l-canavanine and compared their viability and their fecundity to flies maintained on medium without l-canavanine (*n* = 8). When flies fed on 10 mM l-canavanine, we did not observe massive mortality or dramatic decrease of the lifespan. However, all the offspring of flies constrained to eat 10 mM l-canavanine died during larval stages (number of offspring in control medium >1,000, number of offspring in 10 mM l-canavanine = 0). These results indicate that *Drosophila* is a l-canavanine–susceptible insect.

### L-Canavanine Mediates a Behavioral Avoidance Response

**Figure 2:**
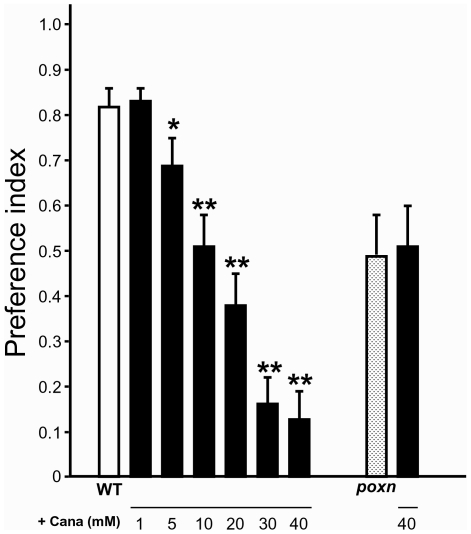
L-Canavanine triggers a chemosensory repulsive effect. Two-choice feeding tests allowed the measure of the preference between the blue and the red solutions of wild-type (WT) and chemosensory defective *pox-neuro* (*poxn*) mutant flies. A preference index (PI) value of 1 or 0 indicates a complete preference or aversion for the blue solution, respectively. A value of 0.5 indicates no preference. See Materials and Methods for the calculation of the PI. In our conditions, WT flies prefer the blue solution (white). When l-canavanine (Cana, black) was added at the indicated concentration (1 to 40 mM), it inhibited the intake of the blue solution in a concentration-dependent manner, being very aversive at 30–40 mM. In contrast, *poxn* flies fed equally on the blue solution with (black) or without (dotted) 40 mM l-canavanine, indicating a chemosensory action for the plant amino acid. Error bars indicate SEM. The single and double asterisks indicate significant differences by *t*-test (*p*<0.05 and *p*<0.001, respectively) between WT flies that fed on the blue solution without (white) or with (black) l-canavanine.

Because of its toxicity, we hypothesized that *Drosophila* may avoid eating l-canavanine if they have the choice. To test this, we performed a two-choice feeding preference test. This behavioral assay measures the consumption of a sucrose solution (5 mM) colored by two food dyes (blue or red) offered simultaneously to adult fly populations [29]. After 2 h in the dark, flies are counted on the color dye witnessed in their abdomen. In the control situation, WT flies preferred the blue solution, the preference index (PI) being 0.82±0.04 (Figure 2). We then added increasing concentrations of l-canavanine to the blue solution (1 mM to 40 mM). We found that l-canavanine inhibited the intake of the blue solution (Figure 2), leading to a symmetrical increase in the intake of the red solution (unpublished data). The l-canavanine effect increased with concentration, reaching a plateau at 30–40 mM. At these concentrations, the PI for the blue solution is 0.13±0.06 (Figure 2). A similar repulsive effect of l-canavanine was also visible when the drug was added to the red solution (unpublished data). To determine whether this repulsive effect was mediated by a chemosensory mechanism, we used flies carrying an adult-viable mutant allele of the *pox-neuro* (*poxn*) gene. In homozygous *poxn* flies, external chemosensilla are deleted or transformed into mechanosensilla [30],[31]. *poxn* flies fed equally on the two colored 5 mM sucrose solutions (PI for blue = 0.49±0.09) (Figure 2). When 40 mM l-canavanine was added to the blue solution, their feeding behavior did not differ (PI for blue = 0.51±0.09, Figure 2). Thus, these results show that l-canavanine is a repellent molecule and that *Drosophila* uses a chemosensory mechanism to detect this plant insecticide.

### DmXR, Encoded by the *mtt* Gene, Is Required In Vivo to Detect L-Canavanine

**Figure 3:**
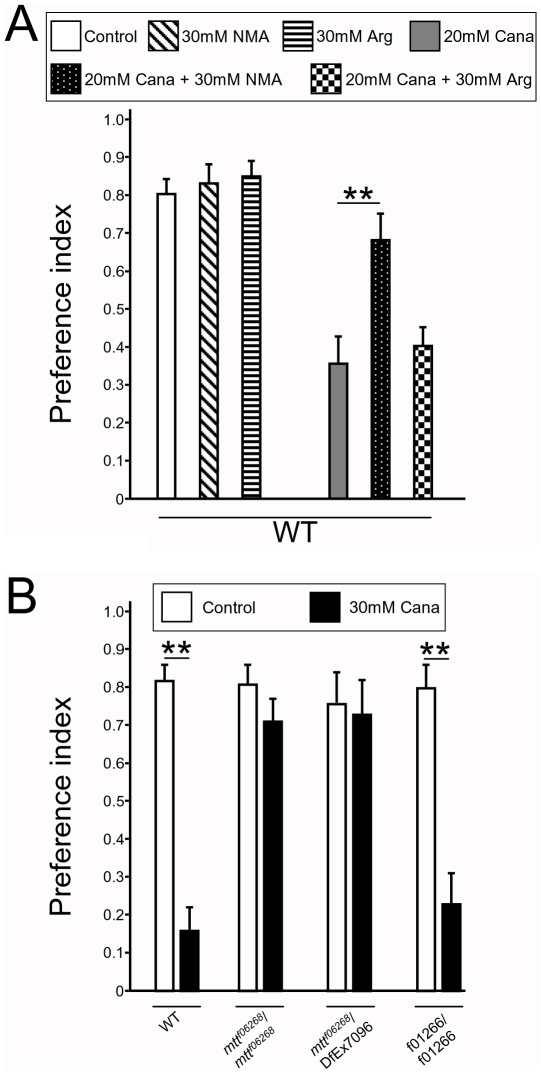
The L-canavanine–induced repulsive behavior requires the DmXR function. (A) Pharmacological inhibition of DmXR by the NMA antagonist reduces l-canavanine repulsive effect. Histograms show the preference index (PI) for the blue solution from two-choice feeding assays in which drugs have been added to the blue solution. There was no significant difference in the behavior of WT flies (WT) that fed similarly on the blue control solution (white) and on the blue solution containing either 30 mM NMA (diagonally hatched) or 30 mM l-arginine (Arg) (horizontally hatched). Significantly more flies fed on the blue solution containing 20 mM l-canavanine (Cana)+30 mM NMA (dotted) than on the blue solution containing 20 mM l-canavanine (grey). l-arginine (30 mM) had no effect on 20 mM l-canavanine repulsive effect (checkered). Error bars indicate SEM. Asterisks indicate significant differences by *t*-test (*p*<0.001). (B) *mtt* mutant flies are insensitive to l-canavanine. Histograms show the preference index (PI) for the blue solution from two-choice feeding assays by using the blue solution without (white) or with (black) 30 mM l-canavanine. WT and the genetic background control *f01266*/*f01266* flies are strongly repulsed by l-canavanine and avoid feeding it. In contrast, *mtt^f06268^/mtt^f06268^* and *mtt^f06268^/DfEx7096* mutant flies are insensitive to l-canavanine, as they fed similarly on the blue solution with or without l-canavanine. Error bars indicate SEM. Asterisks indicate significant differences between the intakes of the blue solution without or with l-canavanine by *t*-test (*p*<0.001).

To study whether DmXR was involved in l-canavanine detection in vivo, we first used a pharmacological approach. We hindered DmXR function by using the NMA antagonist in the two-choice feeding test. When present in the medium, this drug should diminish the repulsive action of l-canavanine. We first tested whether NMA alone could influence the fly feeding behavior: WT flies were allowed to choose between a blue solution containing 30 mM NMA versus a red control solution, both containing 5 mM sucrose. We found that flies were insensitive to 30 mM NMA (PI for blue = 0.80±0.04 and 0.83±0.04 in control and NMA-fed flies, respectively, Figure 3A). However, significantly more flies fed on 20 mM l-canavanine in presence of 30 mM NMA (PI for blue = 0.68±0.07) than on 20 mM l-canavanine alone (PI for blue = 0.36±0.07) (Figure 3A). l-Arginine (30 mM), which is inactive on DmXR, had no effect on l-canavanine-induced repulsive behavior (PI for blue = 0.41±0.05, Figure 3A). Thus, blockade of DmXR with the antagonist NMA lowered the repellent effect of l-canavanine.

**Figure 4:**
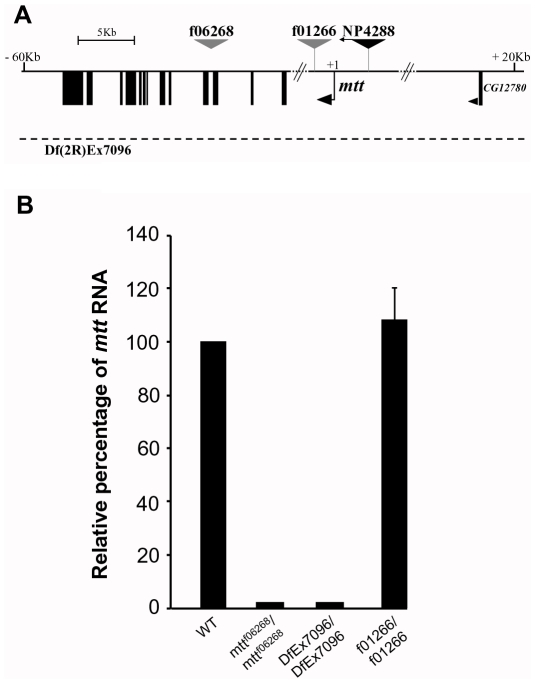
The *f06268* and the *DfEx7096* lines are *mtt* null mutants. (A) Schematic diagram showing the structure of the *mtt* gene and the insertion sites of the transposon lines used in this study. Black boxes show coding regions of *mtt* and CG12780 cDNAs. Black arrow shows the most distal transcription start site (+1) of *mtt* (predicted by Flybase). Arrowhead indicates the orientation of transcription of the CG12780 gene. The *f06268* line carries a piggyBac transposon inserted 35 bp downstream the third *mtt* exon. The *f01266* piggyBac transposon and the NP4288-GAL4 enhancer trap lines are inserted 1.7 Kb downstream and 3.1 Kb upstream, respectively, from the transcription start site of *mtt*. The *Df(2R)Exel7096* line corresponds to a small deficiency that completely removes the *mtt* locus and some adjacent genes (CG8697 to CG2397). (B) QRT-PCR analysis on *mtt* mutants. The expression levels of *mtt* RNA on homozygous wild-type (WT), *f06268*, *Df(2R)Ex7096*, and *f01266* adults flies were measured by quantitative real-time reverse-transcriptase polymerase chain reaction (QRT-PCR). WT and *f01266* homozygous flies show comparable levels of *mtt* RNA, whereas no amplification is obtained from the *f06268* and the *Df(2R)Ex7096* homozygous lines. Error bar indicates SEM.

We then used a genetic approach, taking advantage of two fly lines in which the DmXR encoding gene that we called *mangetout* (*mtt*) is disrupted. The *f06268* line carries a piggyBac transposon inserted into the *mtt* gene as determined by Exelixis sequence analysis [32], and the *Df(2R)Exel7096* line completely removes the *mtt* locus [33] (Figure 4A). Both mutants are viable in homozygous conditions. We expected that the insertion of a transposon 35 bp downstream from the third exon of *mtt* would disrupt the transcription of the gene. Indeed, *f06268* homozygous flies are *mtt* loss-of-function mutants since no RNA was detected by quantitative real-time reverse-transcriptase polymerase chain reaction (QRT-PCR) in adults (Figure 4B). In homozygous *Df(2R)Exel7096* flies, *mtt* RNA was also not detectable by QRT-PCR (Figure 4B). As a control for genetic background, we used flies homozygous for the *f01266* piggyBac transposon line [32] (Figure 4A), which express normal levels of *mtt* RNA (Figure 4B). In control two-choice feeding tests without l-canavanine, homozygous *mtt^f06268^*, hemizygous *mtt^f06268^/Df(2R)Exel7096*, and homozygous *f01266* flies behaved as WT (Figure 3B). When 30 mM l-canavanine was added to the blue solution, *mtt^f06268^/mtt^f06268^* and *mtt^f06268^/Df(2R)Exel7096* flies fed on this blue solution (PI for blue = 0.71±0.06 and 0.73±0.09, respectively), whereas WT and *f01266* flies were repulsed (PI for blue = 0.16±0.06 and 0.23±0.08, respectively) (Figure 3B). Thus, *mtt* mutant flies are insensitive to 30 mM l-canavanine.

Both pharmacological and genetic data lead to the conclusion that DmXR is required for the detection of l-canavanine and that its activation hinders the feeding. Since DmXR may be the l-canavanine–sensitive receptor in chemosensory organs, we wondered whether this receptor was actually localized in these organs.

### *mtt* Is Expressed in Gustatory Sensilla

*Drosophila*, like other insects, base their feeding decisions on the presence or absence of specific volatile and nonvolatile chemicals present in the food. Volatile chemicals are in general detected by olfactory neurons, located mainly on the antenna, whereas nonvolatile chemicals like amino acids are detected by gustatory receptor neurons (GRNs). GRNs are present in taste sensilla localized in the legs, the labial palps (or labellum) found on the tip of the proboscis, and within the pharynx (called internal taste organs) [34]. As flies walk on their food sources, tarsal gustatory sensilla evaluate their chemical contents. If phagostimulants are present, the fly extends its proboscis, enabling labellum sensilla to have contact with the food. In the labellum, gustatory chemosensilla house two to four GRNs as well as a single mechanosensory neuron [35]–[37]. In each sensillum, the different subsets of specialized taste neurons are activated by specific classes of tastants, allowing *Drosophila* to detect sugars, bitter compounds, and water [4],[38].

**Figure 5:**
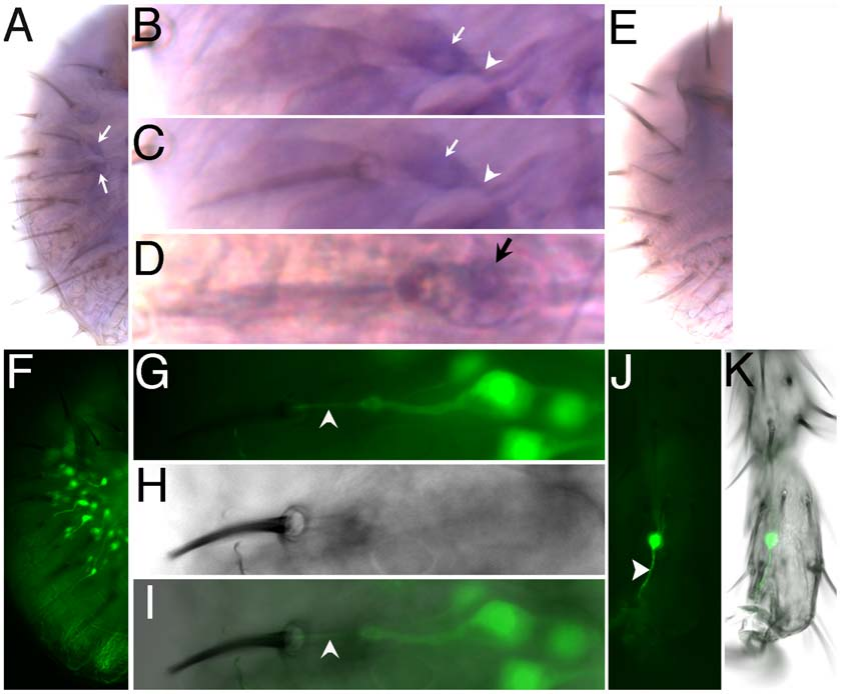
*mtt* is present in chemosensory sensilla. (A–D) and (E) show *mtt* in situ hybridization on WT and *Df(2R)Ex7096 mtt* mutant labellum, respectively. (A) In WT labellum, single cells (arrows indicate two single cells in focus) are labeled by the *mtt* riboprobe. (A) is a composite image of one image focusing on the labeled cells and one image focusing on the chemosensory bristles. (B) and (C) are high-magnification views of one sensilla visible in (A) (upper arrow). (B) focuses on the labeled cell. (C) is a composite image of (B) and a different focal plane in which the nearby chemosensory bristle is visible. Note that one single neuron-like cell (indicated by an arrow) is labeled near the chemosensory bristle visible in (C). This cell is likely a neuron, as an axon is weakly visible (indicated by an arrowhead). (D) shows another sensilla with a different Nomarski setting illustrating that the single labeled cell (indicated by a black arrow) is present in a chemosensilla housing at least five cells. (E) No signal is detected on *Df(2R)Exel7096* mutant labellum. (E) is a composite image of one image focusing within the labellum and one image focusing on the chemosensory bristles. (F–I) Labellum of *NP4288-GAL4/UAS-nlsGFP* showing the GFP-expressing taste neurons. (F) In one labial palp, around 28 GRNs are present (see also Figure S3). (I) is a composite image of the fluorescent and bright-field images shown in (G) and (H), respectively. Arrowhead in (G and I) indicates the dendrite innervating the taste bristle sensilla. (J and K) *NP4288-GAL4/UAS-mCD8GFP* double homozygous foreleg showing that taste neurons are labeled in the tarsi. Arrowhead in (J) indicates the dendrite of the taste neuron. Note that only one of the two labeled neurons is visible at this focal plane. (K) is a composite image of the fluorescent and bright-field images.

To investigate whether *mtt* is expressed in gustatory sensilla, we first performed QRT-PCR experiments on dissected labellum and tarsi/tibiae of WT flies. As shown in Figure S2, *mtt* RNA is expressed in both organs bearing gustatory sensilla. To assess whether *mtt* would be present in GRNs, we compared the expression level of *mtt* RNA in WT to that found in *poxn* mutants, where chemosensory neurons are transformed into mechanosensory neurons. A significant decrease of the amount of *mtt* RNA was detected in the labellum and the tarsi/tibiae of *poxn* mutant compared to WT (Figure S2), suggesting that *mtt* is expressed in some GRNs. To visualize whether *mtt* is indeed expressed in gustatory chemosensilla, we then performed in situ hybridization experiments on labellum of WT flies. We found that *mtt* riboprobe hybridized to a single neuron-like cell within some chemosensory sensilla (Figure 5A–5D). These sensilla, which house five neurons, are clearly gustatory sensilla, because mechanosensory sensilla contain a single, mechanosensory neuron [39]. The in situ labeling appeared to be specific for *mtt* because chemosensory sensilla were not labeled in labellum from *Df(2R)Exel7096* homozygous *mtt* mutant flies (Figure 5E). Altogether, QRT-PCR and in situ hybridization data indicate that *mtt* is expressed in only one GRN per labellar chemosensillum, consistent with a role of DmXR as a taste receptor.

Due to its very low level of expression, we were unable to use the in situ hybridization technique combined with immunocytochemistry to further analyze the *mtt* expression pattern. Hence, in order to investigate the nature of the GRNs expressing *mtt*, we took advantage of a GAL4 enhancer trap line, *NP4288-GAL4*, inserted 3.1 Kb upstream from the transcription start site of *mtt* (Figure 4A). A similar strategy had been undertaken for many other Grs, also reported to have a low level of expression [40]. The expression patterns of these receptors were analyzed using GAL4 transgenes containing taste receptor promoters [29],[40]–[42] or enhancer traps such as NP1017 [43]. These studies have shown that *Gr66a-GAL4*, *Gr5a-GAL4*, and *NP1017-GAL4* lines drive specific expression in bitter-, sugar-, and water-sensitive GRNs, respectively [29],[42],[43]. When *NP4288-GAL4* was crossed with a green fluorescent protein (GFP) reporter line, we observed GFP-positive neurons in taste organs. In the labellum, chemosensory sensilla contained one GFP-positive neuron (Figure 5F–5I), in accordance with the in situ hybridization data. We observed around 28 GFP-positive neurons per labial palp, and two in each tarsus of the legs (Figure 5J and 5K, and Table 1). In addition, four GFP-positive neurons were present in the labral sense organs (LSO) and in the ventral cibarial sense organs (VCSO) (unpublished data), which are bilaterally symmetrical internal taste organs located in the pharynx [44]. Interestingly, *Gr66a-GAL4*, but not *Gr5a-GAL4* or *NP1017-GAL4*, drives also expression in the LSO and VCSO (Table 1), suggesting that *NP4288* is expressed in bitter-sensitive GRNs. To determine whether *NP4288-GAL4* and *Gr66a-GAL4* are indeed coexpressed, we analyzed transgenic fly lines expressing *UAS-nlsGFP* under the control of both *NP4288* and *Gr66a GAL4* drivers, and then counted and compared the number of GFP-positive neurons to that of flies containing each driver alone. In flies that express either the *NP4288-GAL4* or *Gr66a-GAL4* driver, an average of 28.4 and 26.6 neurons were observed per labial palp, respectively (Figure S3). In flies that express both drivers, an average of 28.8 neurons were detected per palp (Figure S3). Furthermore, we also observed coexpression of both drivers in the LSO and in the foreleg tarsi (table in Figure S3). This indicates that most, if not all, GRNs that express *Gr66a* also express *NP4288,* suggesting that this transgene reflects a role for DmXR in bitter-sensitive GRNs. Altogether, these results show that *mtt* is expressed in one GRN per sensilla that likely correspond to the bitter-sensitive GRNs.

**Table 1:** Expression patterns of the GAL4 lines described in this study.

	Labellum	Internal Taste Sensilla		Legs
		LSO	VCSO	
Gr5a	Yes	No	No	Yes
Gr66a	Yes	Yes	Yes	Yes
NP1017	Yes	No^a^	No^a^	No
NP4288	Yes^a^	Yes^a^	Yes^a^	Yes^a^

To analyze the expression of NP1017 and NP4288 driver lines, we crossed each of them with UAS-nlsGFP flies to visualize the presence of GFP-positive neurons. Also included are observations made by the groups that originally characterized the expression pattern of Gr5a, Gr66a, and NP1017 drivers [29],[40],[43],[60].

a This study.

### Leg GRNs Are Sensitive to L-Canavanine via DmXR

**Figure 6:**
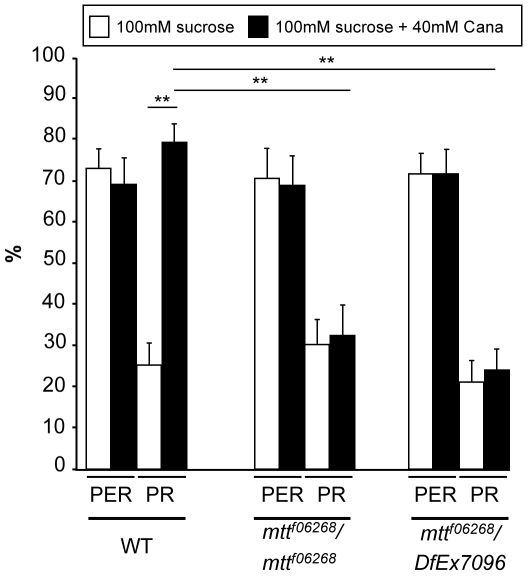
L-Canavanine does not affect the occurrence of PER, but induces a DmXR-dependent retraction of the proboscis after the reflex. For behavioral analyses, the solutions (100 mM sucrose in white and 100 mM sucrose+40 mM l-canavanine in black) were put in contact with the leg tarsi during the 5-s assay. l-Canavanine (Cana) does not affect the percentage of proboscis extension reflex (PER) in WT and *mtt* mutant (*mtt^f06268^/mtt^f06268^* and *mtt^f06268^/DfEx7096*) flies. After the PER response, l-canavanine triggers the proboscis retraction (PR) in WT, but not in *mtt* mutant flies. Error bars indicate SEM. Asterisks indicate significant differences by t-test (*p*<0.001).

To investigate whether GRNs are sensitive to l-canavanine, we examined a direct behavioral measure of leg GRN stimulation by the proboscis extension reflex (PER) paradigm: when the leg tarsi encounter an attractive sugar solution, the proboscis often extends [42],[43],[45]. If a toxic or bitter compound is added to the sugar solution, the PER is inhibited [42],[43],[45]. This assay enables the application of the drugs only on the legs, which carry solely taste sensilla. In addition, we took care that the proboscis never touched the drugs when it extended, so that we were sure that there was no ingestion of drugs (and consequently, no central effect of these drugs). Using the classical PER paradigm (5-s stimulation by touching the leg tarsi either with a 100 mM sucrose solution or with a 100 mM sucrose+40 mM l-canavanine solution), we found that the occurrence of PER was not affected by l-canavanine in WT or *mtt* mutant flies (Figure 6). However, after the PER, WT flies usually sustain their proboscis extension to search for food when their legs are maintained in contact with the sugar solution as shown in Video S1. This sustained phase was strongly affected by l-canavanine, since significantly more flies prematurely retracted their proboscis (78±5% of proboscis retraction [PR]) compared to 25±4% of PR for the sucrose solution, Figure 6). This PR phenotype occurred generally just after the proboscis extension (Video S1). We then tested whether the l-canavanine–induced PR phenotype requires DmXR and found that this phenotype disappeared in the *mtt* loss-of-function mutants (around 25% of PR, Figure 6). Altogether, these data indicate that DmXR is required in leg GRNs for the l-canavanine detection.

### DmXR Is Required in Bitter-Sensitive GRNs for L-Canavanine Sensitivity

**Figure 7:**
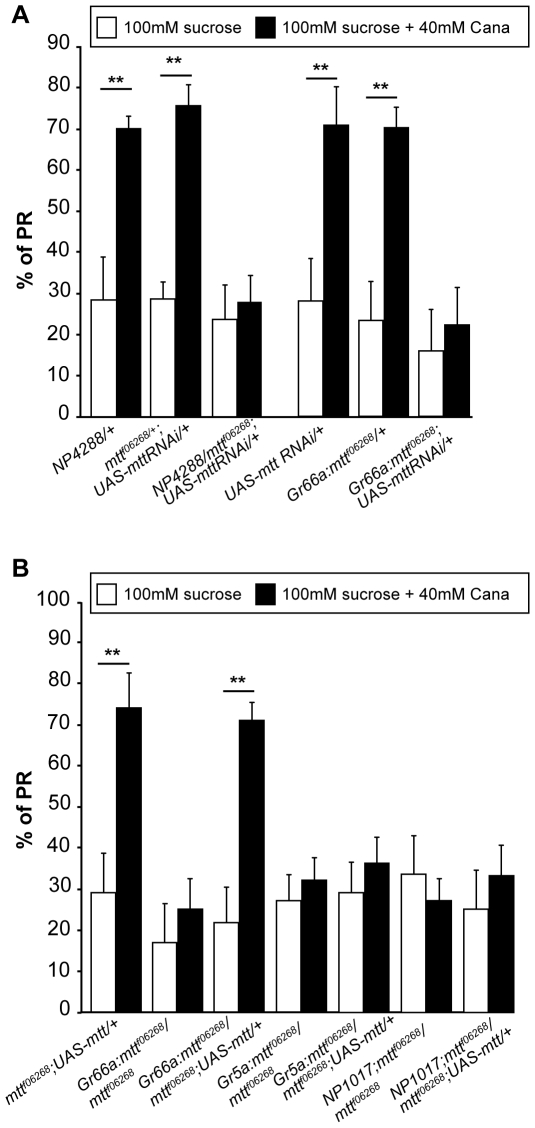
*mtt* is required in Gr66a-GRNs. (A) Knockdown of *mtt* expression by RNAi in NP4288-GRNs and Gr66a-GRNs suppresses the l-canavanine (Cana)-induced PR gustatory phenotype. Histograms show the percentage of PR of controls (*NP4288/+*, *mtt^f06268^/+;UAS-mtt RNAi/+*, *UAS-mtt RNAi/+*, and *Gr66a:mtt^f06268^/+*) and *mtt* heterozygous flies expressing the *mtt* RNAi construct in NP4288-positive GRNs (*NP4288/mtt^f06268^;UAS-mtt RNAi/+*) or in Gr66a-GRNs (*Gr66a:mtt^f06268^;UAS-mtt RNAi/+*). Compared to controls, the down-regulation of *mtt* in NP4288-GRNs or in Gr66a-GRNs suppresses the l-canavanine–induced PR. For all genotypes, l-canavanine does not significantly affect the percentage of PER (see Figure S4A). Behavioral analyses were performed as described in Figure 6. Error bars indicate SEM. Double asterisks indicate significant differences by *t*-test (*p*<0.001). (B) Expression of *mtt* in bitter-sensitive Gr66a-GRNs rescues the PR mutant gustatory phenotype of *mtt* mutant flies. Histograms show the percentage of PR of control flies (*mtt^f06268^/+;UAS-mtt/+*), *mtt* mutant flies carrying one copy of each GRN GAL4 (*Gr66a:mtt^f06268^/mtt^f06268^*, *Gr5a:mtt^f06268^/mtt^f06268^*, and *NP1017/+;mtt^f06268^/mtt^f06268^*) and *mtt* mutant flies expressing *mtt* in bitter-, sugar-, and water sensitive GRNs (*Gr66a:mtt^f06268^/mtt^f0^* ^6268^;UAS-mtt/+, *Gr5a:mtt^f06268^/mtt^f06268^;UAS-mtt/+*, and *NP1017/+;mtt^f06268^/mtt^f06268^;UAS-mtt/+*, respectively). Note that only *mtt* mutant flies expressing *mtt* in Gr66a-GRNs show a rescue of the l-canavanine–induced PR phenotype.

To determine in which GRNs *mtt* is required, we established transgenic flies carrying a *mtt* RNA interference (RNAi) construct under the control of UAS sequence [46]. We first expressed *mtt* RNAi with *NP4288-GAL4* in heterozygous *mtt^f06268^* flies and tested the effect of l-canavanine by the PER/PR behavioral assay. The occurrence of PER was not affected by l-canavanine in controls and *mtt*-knockdown flies (Figure S4A). However, RNAi knockdown of *mtt* suppressed the l-canavanine–induced premature PR phenotype (Figure 7A) in a comparable manner to that observed in *mtt* mutant flies. This indicates that *mtt* is expressed in *NP4288-GAL4*–positive cells, which overlap Gr66a-GRNs in taste organs. However, *NP4288-GAl4* drives also expression in cells during development and in the adult brain (unpublished data), precluding us from concluding that *mtt* is required only in GR66a-GRNs for l-canavanine detection. Thus, we next used the *Gr66a-GAL4* driver to specifically express the *mtt* RNAi in Gr66a-GRNs of heterozygous *mtt^f06268^* flies. As already observed with *NP4288-GAL4*–driven knockdown of *mtt*, the occurrence of PER was unmodified in this genetic condition (Figure S4A). Importantly, *Gr66a-GAL4*–induced knockdown of *mtt* significantly reduced the occurrence of l-canavanine–induced premature PR (Figure 7A). This clearly indicates a requirement of *mtt* in Gr66a-GRNs for l-canavanine sensitivity.

To clearly demonstrate that l-canavanine detection required the presence of DmXR only in Gr66a GRNs, we performed rescue experiments by targeting *mtt* expression in distinct types of GRNs of *mtt* homozygous mutants by using the GAL4/UAS system [46]. Indeed, several types of GRNs are present in the tarsi, such as sucrose-, bitter-, and water-sensitive GRNs (Figure S5). Expression of *mtt* in the different subsets of GRNs did not affect the percentage of PER (Figure S4). We then analyzed the PR and found that expression of *mtt* in sugar or water GRNs did not rescue the mutant phenotype (Figure 7B). In contrast, expression of *mtt* in Gr66a-GRNs rescued l-canavanine sensitivity (Figure 7B). Thus, it is the stimulation of DmXR, in Gr66a-GRNs, which is responsible for l-canavanine–induced PR. To further verify that only Gr66a-GRNs are necessary for l-canavanine sensitivity, we expressed the *hid* and *rpr* proapoptotic genes [47] in these neurons, or inhibited their neurotransmitter release with the tetanus toxin transgene [48]. In both cases, the PER was not affected (Figure S6), but the l-canavanine–induced PR was lost (Figure S6). Altogether, these results demonstrate that DmXR is expressed and required only in Gr66a-GRNs for l-canavanine detection.

### DmXR Is a Gustatory Receptor

**Figure 8:**
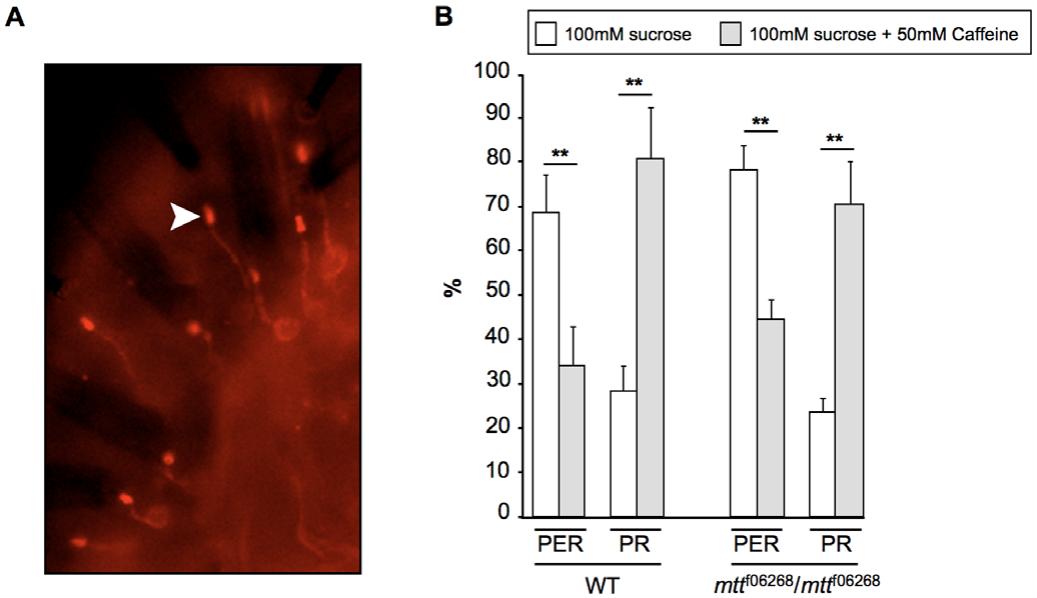
DmXR is localized to GRN dendrites and is dispensable for caffeine sensitivity. (A) Subcellular localization of the DmX receptor. Anti-HA immunostaining on labellum of *Gr66a-GAL4/+;UAS-HAmtt/+* flies. HAmtt is a HA-tagged version of the DmX receptor. When DmXR is expressed in Gr66a-GRNs, it is principally targeted to the dendrite. Note the strong localization of the receptor at the tip of the dendrite (arrowhead). (B) The aversive effect of caffeine is not affected in *mtt* mutant flies. Behavioral analyses were performed as described in Figure 6 except that 100 mM sucrose (white) and 100 mM sucrose+50 mM caffeine (grey) solutions were used. Histograms show the percentage of PER and PR of control (WT) and *mtt* mutant (*mtt^f06268^/mtt^f06268^*) flies. For both control and *mtt* mutant flies, the caffeine inhibits the sucrose-induced PER. This effect is not complete as some flies still performed a PER in the presence of caffeine. Most of these flies retract their proboscis in response to caffeine, similar to what is observed with l-canavanine. Error bars indicate SEM. Double asterisks indicate significant differences by *t*-test (*p*<0.001).

The sites of taste reception are localized to the dendrites of GRNs [36], which are bipolar neurons containing a single dendrite and a single axon. To confirm that DmXR was actually the l-canavanine taste receptor and not a regulatory receptor modulating Gr66a-GRN synaptic transmission, we performed two kinds of experiments. First, we expressed a HA-tagged receptor in Gr66a-GRNs to determine its subcellular localization in the labellum. As shown in Figure 8A, the receptor was highly concentrated at the dendrite and not detected in the GRN axon in accordance with a gustatory function. Second, we tested whether DmXR could modify Gr66a activation by other repellents than l-canavanine. It has been shown that Gr66a-GRNs are required for caffeine-aversive behavior, Gr66a being a gustatory receptor for caffeine [8]. As already published, we could find that caffeine inhibited sucrose-induced PER (Figure 8B). However, when the PER occurred, we noticed that there was a high rate of premature PR. The caffeine-induced PR phenotype occurred generally just after the PER, similar to what was observed with l-canavanine (Video S2). We then tested the caffeine-induced phenotypes in *mtt* mutants and did not find changes in caffeine-induced PER inhibition and caffeine-induced PR (Figure 8B). These data clearly demonstrate that DmXR acts as a l-canavanine gustatory receptor in Gr66a-GRNs.

## Discussion

The ability to avoid ingestion of toxic plants compounds is crucial for insect survival. However, before the current study, only two receptors, Gr66a and Gr93a, which are essential for the caffeine response, were associated with a specific bitter tastant [7],[8]. The nonprotein amino acid l-canavanine is known to be toxic to insects, when ingested ([18] and this study). Here, our results show that l-canavanine is detected as a repulsive molecule. With a pharmacogenetic approach, we have shown that *Drosophila* uses a taste detection mechanism mediated by the orphan GPCR, DmXR, which is activated by l-canavanine to trigger this avoidance behavior. This process occurs in bitter-sensitive GRNs where this receptor is expressed.

By using the two-choice feeding test, the repulsive effect of l-canavanine was clearly demonstrated. Contrary to the known repellents (caffeine, quinine [8],[49]), l-canavanine does not affect the PER. However, l-canavanine triggers the retraction of the proboscis following its initial extension impairing the food intake. Indeed, after the PER, WT flies usually sustain their proboscis extension to search for food when their legs are maintained in contact with the sugar solution. This sustained phase is strongly affected by l-canavanine, since significantly more flies prematurely retracted their proboscis at this stage. This inhibition of sustained proboscis extension is not specific for l-canavanine, but is also observed, with the same rate, in the presence of caffeine. Hence, caffeine induces a fast response, which is the PER inhibition, and a slow response, which is the PR, whereas l-canavanine only induces the slow response. One open question is to understand the molecular mechanisms responsible for such a difference between caffeine and l-canavanine in the response dynamics, knowing that both drugs act on the same cell type (Gr66a-GRNs). GPCR-induced signal transduction pathways rely on the activation of intracellular heterotrimeric G-proteins [50]–[52]. To date, two G-proteins have been implicated in the taste pathway, and both are required for sugar perception [53],[54]. However, a direct evidence for a coupling between Grs and G-proteins has not been demonstrated [38]. As we have shown in our cell transfection assay ([27] and this study), DmXR is a genuine GPCR. It is thought that the Gr66a receptor, like other Grs, is a putative GPCR [8],[38]. However, we do not know to which type of G-protein these two receptors are coupled in vivo. A first explanation about the different dynamics induced by l-canavanine and caffeine could be that that DmXR and Gr66a are coupled to distinct G-proteins or that both receptors are coupled to the same G-protein, but with different efficiencies. A more speculative explanation may be due to the different structural features of the two receptors, taking into account that Grs are structurally related to the olfactory receptors in *Drosophila* [40]. As was recently shown, *Drosophila* olfactory receptors may act as ligand-gated channels instead of being coupled to a G-protein [55],[56]. Thus, Gr66a receptor may also be a ligand-gated channel. Because of the absence of any intermediate, changes in membrane excitability would be more rapid in presence of caffeine compared to l-canavanine. This may explain the difference in the response dynamics between these two drugs.

This study shows that DmXR is expressed and required in bitter-sensitive leg GRNs. However, DmXR is also known to be expressed in the adult brain, in agreement with our observations that NP4288-GAL4 is expressed in this tissue (unpublished data). This suggests the existence of an unknown endogenous ligand, different from l-canavanine, triggering DmXR activation in the brain. To exclude any action of l-canavanine in the brain, we took care that flies avoided ingesting the drug solutions by applying them only on legs during the PER analysis. In addition, we used GRN-restricted drivers, allowing us to specifically analyze the peripheral function of DmXR. Finally, the absence of any defects in the caffeine-induced response of *mtt* mutants flies excludes a role of DmXR in second and higher order neurons involved in the control of the studied gustatory behavior. As we observed *mtt* expression in the labellum and in internal taste organs (LSO and VCSO), it is very likely that these taste sensilla also play a role in the l-canavanine–induced aversive behavior. In agreement with this, we observed that flies did not drink a l-canavanine/sucrose–containing solution when directly applied on the labellum (unpublished data), confirming the presence of l-canavanine–sensitive GRNs. So, we assume that DmXR is a l-canavanine–tuned gustatory receptor in all these taste organs.

Surprisingly, it is not a Gr member that has been selected to detect l-canavanine, despite the very high sequence diversity of this family. Indeed, DmXR belongs to the mGluR GPCR subfamily because of its close sequence relationship [27]. DmXR and mGluR LBP sequences and 3-D model comparisons have shown that DmXR has diverged only in the LBP part interacting with the γ-carboxylic group of glutamate [27]. These modifications have targeted and changed two residues that are conserved in all mGluRs and are crucial for glutamate-induced activation [24],[27]. Our study shows that these structural changes are correlated with the ligand selectivity of the receptor. Indeed, DmXR has a divergent LBP so that glutamate is no more an agonist but l-canavanine is. Conversely, the *Drosophila* mGlu ortholog receptor, DmGluA, is not activated by l-canavanine. This suggests that the original conformation of the mGluR LBP was more adapted to diverge and to recognize l-canavanine than the one of the Grs. In addition, to give rise to this new type of gustatory receptor, appropriate GRN expression has also been added during the evolution of the DmXR function since mGluR expression is mainly found in the central nervous system [25]. Thus, our study suggests that other GPCRs, different from Grs, may have evolved in insects to recognize specific tastants.

Finally, the insect-borne diseases are largely increasing, partly due to the development of insecticide resistance. Thus, it becomes urgent to identify insect-specific targets for the design of new drugs against insects. Our work illustrates that the pharmacological and functional characterization of the insect-specific GPCRs, which likely control insect-specific physiological processes, is a way to discover new protection or fighting strategies against harmful insects.

## Materials and Methods

### Chemicals and Pharmacology

l-glutamate, l-canavanine, l-arginine, and γ-*N*-methyl-l-arginine (NMA) were from Sigma. Human embryonic kidney (HEK 293) cells were cultured and transiently transfected by electroporation as previously described [27]. Carrier plasmid DNA (pRK5) (14 μg), plasmid DNA containing *HA-DmXR WT, HA-DmXRT176A* mutant (4 µg), DmGluRA (2 µg), and plasmid DNA containing Gαqi9 (2 µg) (to enable the artificial coupling of DmXR and DmGluRA to phospholipase C, [57]) were used for the transfection of 10^7^ cells. Determination of inositol phosphate (IP) accumulation in transfected cells was performed as previously described [27].

### Genetics, Fly Lines, and Constructs

*Drosophila* stocks were raised on standard fly food medium at 25°C on a 12-h light/dark cycle. WT Canton S flies were used as control flies in all behavioral assays. For experiments using *pox-neuro* (*poxn*) adult mutant flies, homozygous flies carrying the *poxn^70−23^* mutant allele were used [30]. *mtt^f06268^* and *f01266* lines carry PBac(WH) transposon and are described in [32]. The *Df(2R)Exel7096* line carries a small deficiency that completely removes the *mtt* locus and some adjacent genes (CG8697 to CG2397) [33]. The p(*UAS-mtt*) transgene construct was generated by cloning the hemagglutinin N-terminally tagged full coding sequence of DmXR (HA-*mtt*) [27] into the pUAST transformation vector and injected into *w^1118^* embryos. Several insertions lines were obtained. After QRT-PCR analysis (unpublished data), the *w^1118^;UAS-mttC5* line was chosen for this study. The *mtt* RNAi line was obtained after amplifying DmXR cDNA sequence with the sense primer 5′-ACT ACT TCT AGA GGC GAT GTG GCA ACA G-3′ and the antisense primer 5′-CCG GGC TCT AGA ATA AGT TTG TTT GCA G-3′. This sequence was digested with the XbaI restriction enzyme and subcloned into AvrII-digested pWIZ. This new construct was then digested with the NheI restriction enzyme and ligated with the same XbaI-digested PCR product. A clone with the second insert oriented opposite to the first was then selected and used for injection of *w^1118^* embryos. Several insertion lines were obtained. After QRT-PCR analysis (unpublished data), the *w^1118^;UAS-mtt RNAi1* line was chosen for this study. The *Gr66a-GAL4* (II chromosome) and *Gr5a-GAL4* (II chromosome) promoter *GAL4* lines are generous gifts from H. Amrein (Duke University, United States). The *NP1017-GAL4* (X chromosome) enhancer trap line was kindly provided by T. Tanimura (Kyushu University, Japan). The NP4288 enhancer trap was obtained from the GETDB Stock Center (Kyoto, Japan) [58]. The *UAS-hid:UAS-rpr* line was a gift from J. R. Martin (Paris Sud University, France). The UAS-TeT line was kindly provided by C. J. O'Kane (Cambridge University, England). The *w;UAS-mCD8-GFP* and the *w;UAS-nlsGFP* were obtained from the Bloomington Stock Center.

### Behavioral Assays

#### Two-choice feeding preference test

The two-choice feeding preference test was performed as described elsewhere, with minor modifications [29]. To enhance the feeding activity during the tests, we used a 5 mM sucrose solution instead of 1 mM. At this concentration of sucrose, WT flies showed a preference for the blue solution as shown in Figure 1. For each trial, 3- to 5-d-old adult flies (30 males and 30 females) were starved on water-saturated cotton for 24 h. Flies were then placed on a 96-well microtiter plate at 23°C to 25°C in the dark. Wells contained 5 mM sucrose test solutions in 0.3% agarose with 5 mg/ml erioglaucine dye (blue) or 20 mg/ml sulforhodamine B dye (red) in the alternating wells, both at pH 7.5. Sucrose, erioglaucine, and sulforhodamine B were from Sigma. After 2 h on the plates, the numbers of flies that were blue (*N* ^B^), red (*N* ^R^), or purple (*N* ^P^) were determined on the basis of the colors of the abdomen. The preference index (PI) values for the blue solution were calculated according to the following equation: (*N* ^B^+0.5*N* ^P^)/(*N* ^B^+*N* ^P^+*N* ^R^). In all the tests shown, the drugs (pH 7.5) were added to the blue solution. Similar results were obtained when l-canavanine was added to the red solution (unpublished data). At least eight independent trials were carried out for each point. Only trials in which at least 35% of the flies had fed were included for statistical analysis. The percentage of flies with purple abdomens, having ingested both blue and red food, was approximately invariable in our experiments (15%–25%). All error bars represent the standard error of the mean (SEM). Unpaired Student *t*-tests were used to check for significant differences between the indicated pairs of data.

#### Proboscis extension reflex (PER) and proboscis retraction (PR) tests

The PER was examined principally as described in [42],[43]. The 3- to 6-d-old adult flies were maintained on fresh medium for 1 d. Flies were then starved on water-saturated cotton for 20 h. Before the assay, the tarsi of the flies were touched with a water-saturated 3MM Whatman paper. If the drop of water induced PER, the fly was allowed to intake sufficient water. Each fly was tested during 5 s by touching only the leg tarsi with either a 100 mM sucrose solution or 100 mM sucrose+drug solution. Six to eight batches of 40–60 flies were tested for each solution and each genotype. The occurrence of premature PR was also determined during the assay. The percentage of PER represents the number of flies from a given genotype that showed the PER phenotype divided by the total number of flies. The percentage of PR represents the number of flies from a given genotype that showed the PR phenotype divided by the number of flies that have shown a PER. Unpaired Student *t*-tests were used to check for significant differences between the indicated pairs of data.

### Quantitative RT-PCR (QRT-PCR) Analyses

Total RNAs were extracted from whole adult flies (for the analysis of *mtt* mutants) or dissected labella, tarsi, and tibiae (for the analysis of *mtt* expression) by using Trizol (Sigma). cDNAs were generated from 1 µg of total RNAs treated with DNase I (Ambion) by using random decamers (Ambion) and Moloney murine leukemia virus reverse transcriptase (Invitrogen). Real-time PCR was done using Applied Biosystems SYBR Green PCR mix according to the manufacturer's instructions. PCR was done as follows: 10 min at 95°C followed by 40 cycles: 15 s at 95°C, 60 s at 60°C. Housekeeping genes used to normalize DmXR expression levels were *RpL13*, *Tbp*, and *Pgk*. Sequences of the primers are *RpL13* 5′-AGGAGGCGCAAGAACAAATC and 5′-CTTGCTGCGGTACTCCTTGAG, *Tbp* 5′-CGTCGCTCCGCCAATTC and 5′-TTCTTCGCCTGCACTTCCA, *Pgk* 5′-TCCTGAAGGTCCTCAACAACATG and 5′-TCCACCAGTTTCTCGACGATCT, and DmXR 5′-CGAATGCAACTGGTTCCTTCTC and 5′-TGAGGAAGTACTCCTCGAAC.

### In Situ Hybridization

Labella were dissected from flies and collected in 4% paraformaldehyde in PBS with 0.05% Triton X-100 on ice. After fixation overnight at 4°C, samples were washed 6×10 min in PTX (PBS, 2% Triton X-100) at room temperature. Prehybridization was then done for 2 h at 55°C in hybridization buffer (HB) (50% formamide, 5× SSC, 0.5 mg/ml yeast tRNA, 0.1 mg/ml Salmon Sperm DNA, 0.05 mg/ml heparin, 0.3% Triton X-100). Hybridization was performed overnight at 55°C with digoxigenin-labeled antisense *mtt* riboprobe derived from *mtt* cDNA and prepared according to the manufacturer's instructions (Roche). Washes were performed at 58°C in HB followed by washes in HB/PTX mix (3/1, 1/1, and 1/3, respectively). After blocking in 0.5% Blocking Reagent (Roche) in PTX, samples were then incubated with anti-Dig-AP (Roche) overnight at 4°C. Samples were then washed 6×10 min in PTX. NBT/BCIP mix (Roche) was used to visualize the digoxigenin-labeled probe. Samples were mounted in 90% glycerol.

### Immunohistochemistry

To visualize HA-DmXR protein expression, we dissected the labella from *Gr66a-GAL4;UAS-HAmtt/+*flies from adult head, fixed them overnight in 4% paraformaldehyde in 1× phosphate-buffered saline (PBS), 0.3% Triton X-100. Immunostaining was performed in 1× PBS 3% Triton X-100 and 0.5% Blocking Reagent (Roche). The following antibodies were used: monoclonal rat anti-HA (Roche; 1∶200) and Cy3-conjugated donkey anti-rat (Jackson ImmunoResearch; 1∶500). Samples were mounted in Vectashield. Images were acquired using a Leica microscope and CoolSNAP camera.

## Supporting Information

Figure S1 **Structure of L-canavanine and L-arginine.** l-canavanine (2-amino-4-guanidinooxybutyric acid) is a nonprotein amino acid synthesized by leguminous plants that are members of the Lotoidea, a subfamily of the Leguminosae [13],[14],[16]. l-Canavanine has a structural analogy to l-arginine in that the terminal methylene group of arginine is replaced with oxygen.
(0.56 MB PDF)

Figure S2 **The expression levels of mtt RNA are strongly decreased in poxn mutant tarsi/tibiae and labellum.** The relative RNA expression levels of *mtt* were evaluated in WT and *poxn* mutant flies by QRT-PCR. The RNA extraction was exclusively made from dissected tarsi/tibiae as well as labellum. Normalized gene expression of *mtt* was standardized to the relative quantities of three housekeeping genes (*RpL13*, *Tbp*, and *Pgk*). WT was arbitrarily assigned a value of 100%. Note the strong reduction of *mtt* expression in *poxn* mutant. Error bars indicate SEM. Double asterisks indicate significant differences by *t*-test (*p* < 0.001).
(0.05 MB PDF)

Figure S3 **NP4288-GAL4 and Gr66a-GAL4 drive GFP expression in the same GRNs.** (A) Images showing the distribution of the GFP-positive cells in a labial palp of NP4288-Gal4/UAS-nlsGFP (NP4288), Gr66a-Gal4/UAS-nlsGFP (Gr66a), and Gr66a-Gal4+NP4288-Gal4/UAS-nlsGFP (NP4288+Gr66a) flies.
(B) Table showing the average number of GFP-positive cells counted in taste organs. As was previously done for the analyses of Gr expression [29],[59], we compared the number of GFP-positive cells present in NP4288-GAL4/UAS-nlsGFP (NP4288), Gr66a-GAL4/UAS-nlsGFP (Gr66a), and Gr66a-GAL4+NP4288-GAL4/UAS-nlsGFP (NP4288+Gr66a) taste organs. Note that the number of GFP-positive cells observed in the foreleg tarsi of NP4288-GAL4,UAS-nlsGFP flies is higher than what is observed in NP4288-GAL4,UAS-mCD8GFP homozygous flies (see Figure 5E and 5F). This is likely due to GFP concentration in the nucleus compared to the membrane-targeted GFP. However, we can not confirm that all these cells are neuronal cells because axons and dendrites were not visible in the leg. We also observed that the number of GFP-positive cells was lower in NP4288-GAL4,Gr66a-GAL4,UAS-nlsGFP (average  =  2.6) than in NP4288-GAL4,UAS-nlsGFP forelegs (average number  =  4.5). Although we do not have an explanation for this result, this discrepancy was already observed for Gr5a-related receptors: the number of GFP-positive cells was higher in Gr61a-GAL4,UAS-GFP (average number  =  12) forelegs compared to Gr61a-GAL4,Gr5a-GAL4,UAS-GFP (average number  =  9.8) and to Gr61a-GAL4,Gr64f-GAL4,UAS-GFP (average number  =  9.6) [59].
(2.36 MB PDF)

Figure S4 **The PER is not affected in rescue and loss of function experiments.** (A) Knockdown of *mtt* expression by RNAi did not affect the PER response. Histograms show the percentage of PER of controls (*NP4288/+, mtt^f06268^/+;UAS-mtt RNAi/+*, *UAS-mtt RNAi/*+, and *Gr66a:mtt^f06268^/+*) and *mtt* heterozygous flies expressing the *mtt* RNAi construct either in NP4288-positive GRNs (*NP4288/mtt^f06268^;UAS-mtt RNAi/+*) or in Gr66a-GRNs (*Gr66a:mtt^f06268^;UAS-mtt RNAi/+*). Compared to controls, the down-regulation of *mtt* in NP4288-GRNs or in Gr66a-GRNs did not affect the PER response. Behavioral analyses were performed as described in Figure 6. Error bars indicate SEM.
(B) Expression of DmXR in Gr66a, Gr5a, and NP1017 GRNs of *mtt* mutant flies did not affect the PER response. Histograms show the percentage of PER of controls (*mtt^f06268^/+;UAS-mtt/+*), *mtt* mutant flies carrying one copy of each GRN GAL4 (*Gr66a:mtt^f06268^/mtt^f06268^*, *Gr5a:mtt^f06268^/mtt^f06268^*, and *NP1017/+;mtt^f06268^/mtt^f06268^*) and *mtt* mutant flies expressing *mtt* in bitter-, sugar-, and water-sensitive GRNs (*Gr66a:mtt^f06268^/mtt^f06268^;UAS-mtt/+; Gr5a:mtt^f06268^/mtt^f06268^;UAS-mtt/+*, and *NP1017/+;mtt^f06268^/mtt^f06268^;UAS-mtt/+*, respectively). For all genotypes, l-canavanine did not significantly affect the percentage of PER. Behavioral analyses were performed as described in Figure 6.
(0.05 MB PDF)

Figure S5 **The GRN GAL4 lines drive expression of a GFP reporter in GRNs of the first leg tarsi.** The expression patterns of GRN-GAL4 drivers in the first leg tarsi were visualized by GFP epifluorescence. GFP-positive neurons (arrowheads) are observed from either *Gr5a-GAL4/UAS-mCD8GFP, Gr66a-GAL4/UAS-mCD8GFP* or *NP1017-GAL4/+;UAS-mCD8GFP/+* flies. *Gr5a-GAL4/UAS-mCD8GFP* and *NP1017-GAL4/+;UAS-mCD8GFP/+* are lateral views. *Gr66a-GAL4/UAS-mCD8GFP* is an anterior view.
(0.48 MB PDF)

Figure S6 **Gr66a-GRNs are necessary for L-canavanine-induced PR.** By using the *Gr66a-GAL4* driver, we targeted the expression of the proapoptotic genes (*rpr* and *hid*) and the tetanus toxin light chain (*TeT*) to kill and inactivate Gr66a-GRNs, respectively. Results show the percentage of PER and PR on controls (*UAS-rpr:UAS-hid/+* and *UAS-TeT/+*), ablated (*Gr66a/UAS-rpr:UAS-hid*), and silenced (*Gr66a/UAS-TeT*) Gr66a-GRNs. Note that the PER was not affected in controls as well as when Gr66a-GRNs were ablated or silenced. Compared to controls, which show a high percentage of PR in presence of l-canavanine, the absence or the inactivation of Gr66a-GRNs abolishes the PR response. Behavioral analyses were performed as described in Figure 6. Error bars indicate SEM. Double asterisks indicate significant differences by *t*-test (*p* < 0.001).
(0.06 MB PDF)

Video S1 **L-canavanine triggers the premature proboscis retraction after the PER.** This movie illustrates the behavior of WT fly when their leg tarsi are in contact with either a 100 mM sucrose solution (sucrose) or a 100 mM sucrose + 40 mM l-canavanine (sucrose/canavanine) solution. Soon after the sucrose stimulation, PER occurs, and flies usually sustain their proboscis extension to search for food. When the legs are in contact with the sucrose/canavanine solution, PER occurs normally, but the fly retracts its proboscis just after, leading to the end of food searching. Thus, l-canavanine triggers avoidance behavior by premature PR.
(1.02 MB MOV)

Video S2 **Caffeine and L-canavanine trigger the premature proboscis retraction after the PER.** This movie illustrates the behavior of WT flies when their leg tarsi are in contact with either a 100 mM sucrose + 40 mM l-canavanine (sucrose/canavanine) solution or a 100 mM sucrose + 50 mM caffeine (sucrose/caffeine) solution. Generally, caffeine inhibits the sucrose-induced PER. However, this effect is not complete as some flies extend their proboscis as shown in this video. Similar to the l-canavanine–induced effect, the majority of those flies retract their proboscis just after PER in the presence of caffeine.
(1.03 MB MOV)

## Abbreviations

GPCR: G-protein–coupled receptor
Gr: gustatory receptor
GRN: gustatory receptor neuron
LBP: ligand binding pocket
LSO: labral sense organ
mGluR: metabotropic glutamate receptor
PER: proboscis extension reflex
PI: preference index
PR: proboscis retraction
QRT-PCR: quantitative real-time reverse-transcriptase polymerase chain reaction
RNAi: RNA interference
VCSO: ventral cibarial sense organ
WT: wild-type
