# Supplementary figures and images for: Plant Insecticide L-Canavanine Repels Drosophila via the Insect Orphan GPCR DmX
Source: PLoS Biol. 2009 Jun 30;7(6):e1000147. doi: 10.1371/journal.pbio.1000147 (PMC2695807; doi:10.1371/journal.pbio.1000147)

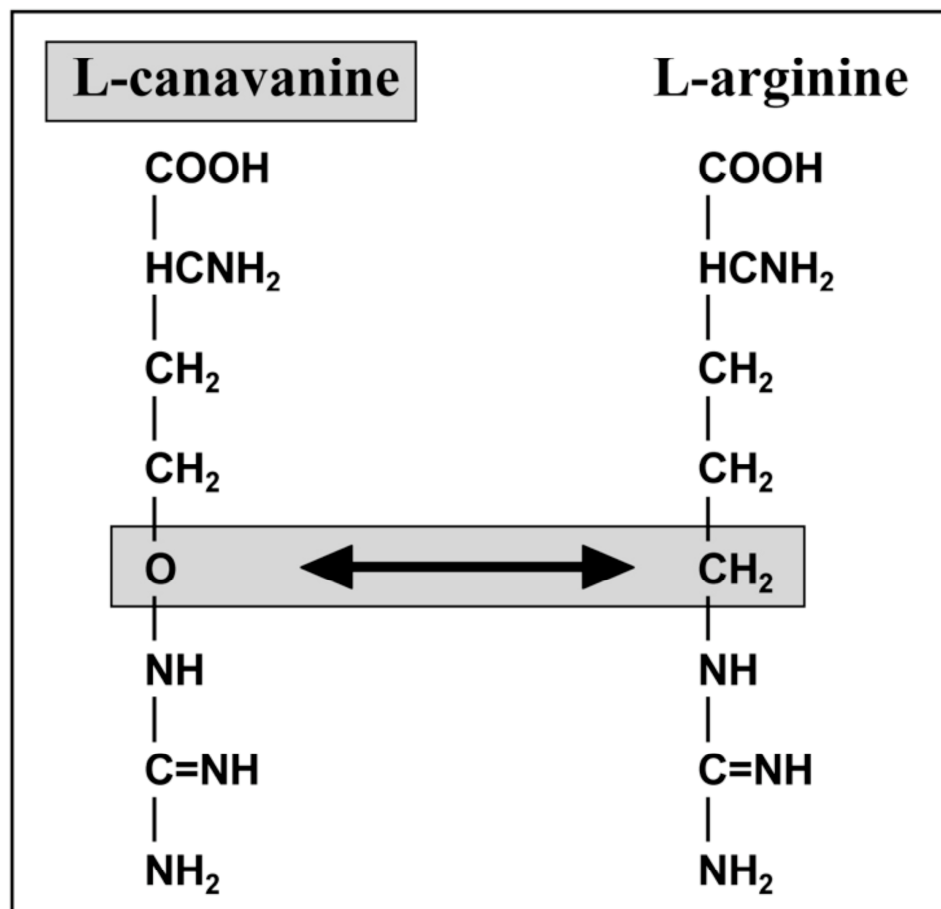

Figure S1

Supplement: Figure S1 — Structure of L-canavanine and L-arginine. l-canavanine (2-amino-4-guanidinooxybutyric acid) is a nonprotein amino acid synthesized by leguminous plants that are members of the Lotoidea, a subfamily of the Leguminosae [13],[14],[16]. l-Canavanine has a structural analogy to l-arginine in that the terminal methylene group of arginine is replaced with oxygen. (0.56 MB PDF) [file pbio.1000147.s001.pdf]

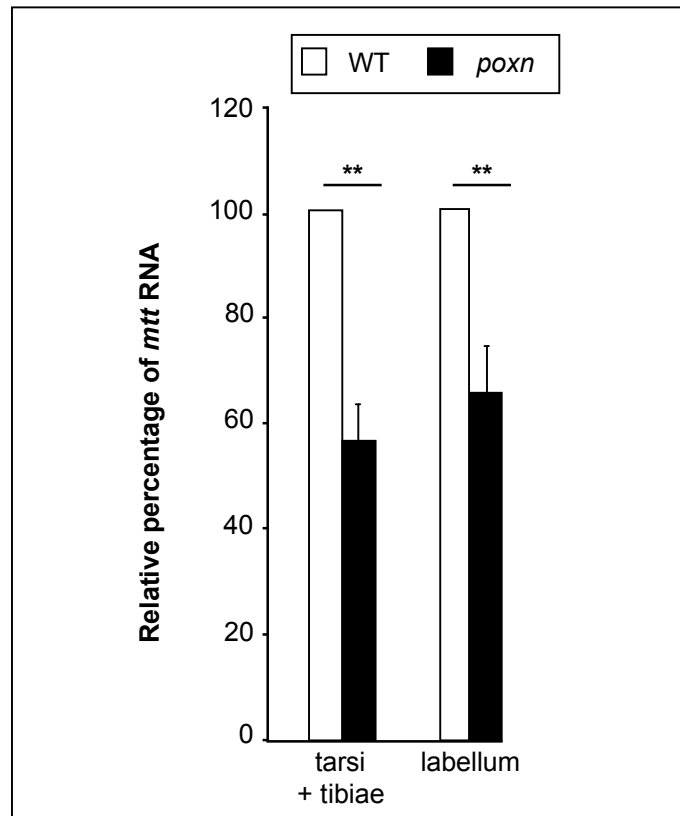

Figure S2

Supplement: Figure S2 — The expression levels of mtt RNA are strongly decreased in poxn mutant tarsi/tibiae and labellum. The relative RNA expression levels of mtt were evaluated in WT and poxn mutant flies by QRT-PCR. The RNA extraction was exclusively made from dissected tarsi/tibiae as well as labellum. Normalized gene expression of mtt was standardized to the relative quantities of three housekeeping genes (RpL13, Tbp, and Pgk). WT was arbitrarily assigned a value of 100%. Note the strong reduction of mtt expression in poxn mutant. Error bars indicate SEM. Double asterisks indicate significant differences by t-test (p < 0.001). (0.05 MB PDF) [file pbio.1000147.s002.pdf]

**A** NP4288

Gr66a

NP4288 + Gr66a

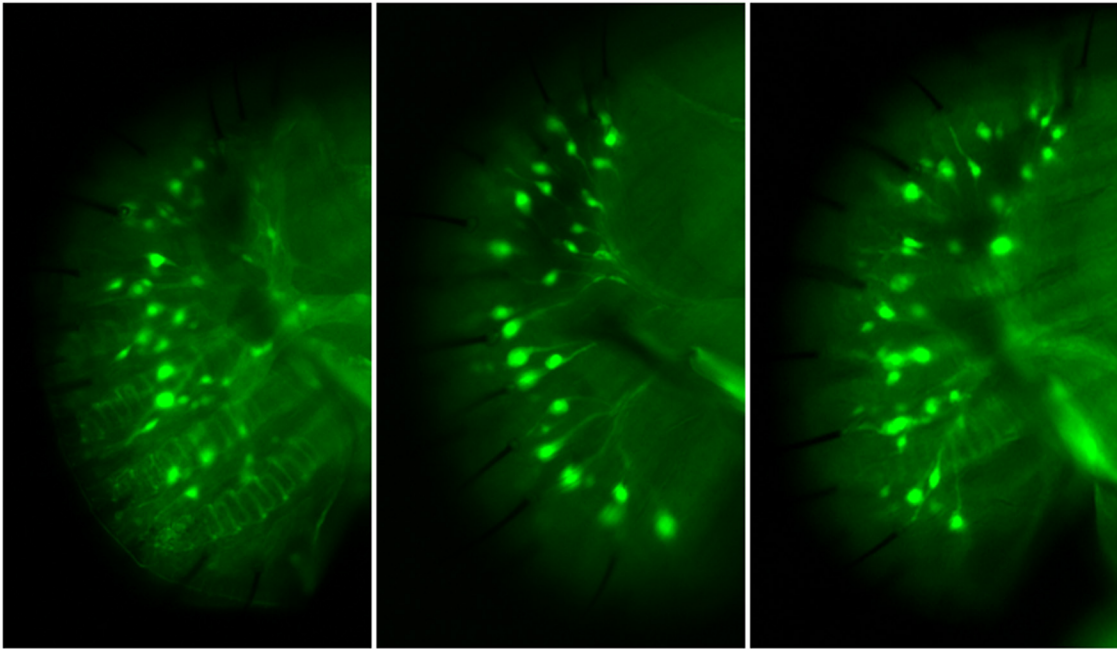

**B**

|               | NP4288 | Gr66a | NP4288 + Gr66a |
|---------------|--------|-------|----------------|
| Labellum      | 28.4   | 26.6  | 28.8           |
| LSO           | 4      | 4     | 4              |
| Foreleg tarsi | 4.5    | 1.9   | 2.6            |

Figure S3

Supplement: Figure S3 — NP4288-GAL4 and Gr66a-GAL4 drive GFP expression in the same GRNs. (A) Images showing the distribution of the GFP-positive cells in a labial palp of NP4288-Gal4/UAS-nlsGFP (NP4288), Gr66a-Gal4/UAS-nlsGFP (Gr66a), and Gr66a-Gal4+NP4288-Gal4/UAS-nlsGFP (NP4288+Gr66a) flies. (B) Table showing the average number of GFP-positive cells counted in taste organs. As was previously done for the analyses of Gr expression [29],[59], we compared the number of GFP-positive cells present in NP4288-GAL4/UAS-nlsGFP (NP4288), Gr66a-GAL4/UAS-nlsGFP (Gr66a), and Gr66a-GAL4+NP4288-GAL4/UAS-nlsGFP (NP4288+Gr66a) taste organs. Note that the number of GFP-positive cells observed in the foreleg tarsi of NP4288-GAL4,UAS-nlsGFP flies is higher than what is observed in NP4288-GAL4,UAS-mCD8GFP homozygous flies (see Figure 5E and 5F). This is likely due to GFP concentration in the nucleus compared to the membrane-targeted GFP. However, we can not confirm that all these cells are neuronal cells because axons and dendrites were not visible in the leg. We also observed that the number of GFP-positive cells was lower in NP4288-GAL4,Gr66a-GAL4,UAS-nlsGFP (average = 2.6) than in NP4288-GAL4,UAS-nlsGFP forelegs (average number = 4.5). Although we do not have an explanation for this result, this discrepancy was already observed for Gr5a-related receptors: the number of GFP-positive cells was higher in Gr61a-GAL4,UAS-GFP (average number = 12) forelegs compared to Gr61a-GAL4,Gr5a-GAL4,UAS-GFP (average number = 9.8) and to Gr61a-GAL4,Gr64f-GAL4,UAS-GFP (average number = 9.6) [59]. (2.36 MB PDF) [file pbio.1000147.s003.pdf]

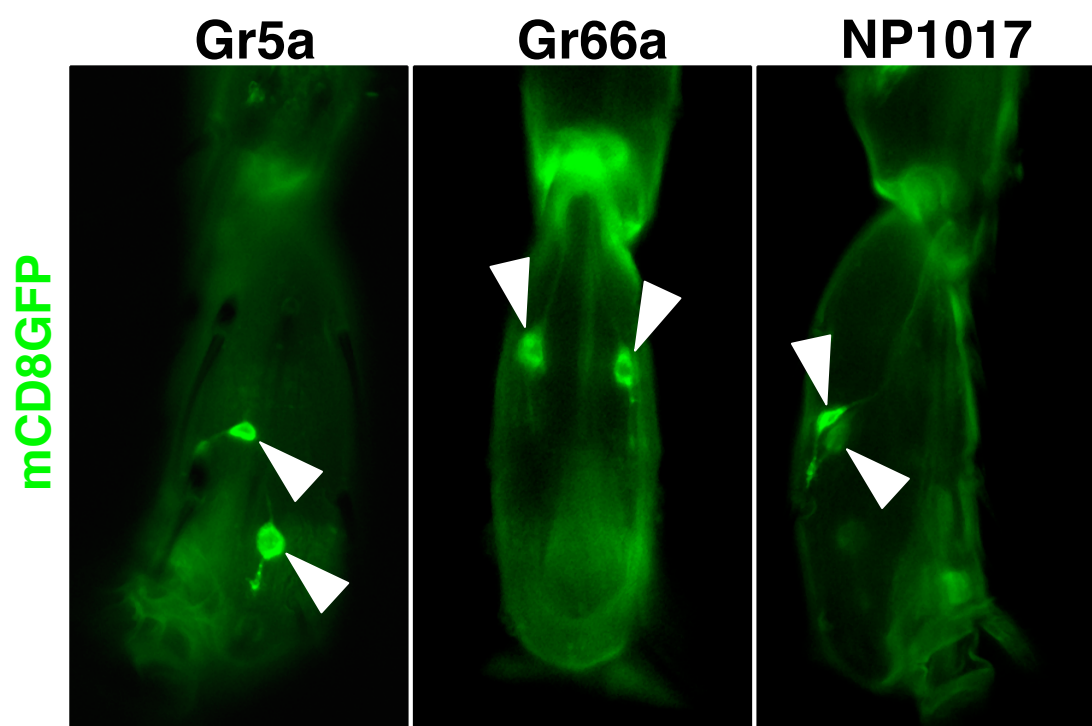

Figure S5

Supplement: Figure S5 — The GRN GAL4 lines drive expression of a GFP reporter in GRNs of the first leg tarsi. The expression patterns of GRN-GAL4 drivers in the first leg tarsi were visualized by GFP epifluorescence. GFP-positive neurons (arrowheads) are observed from either Gr5a-GAL4/UAS-mCD8GFP, Gr66a-GAL4/UAS-mCD8GFP or NP1017-GAL4/+;UAS-mCD8GFP/+ flies. Gr5a-GAL4/UAS-mCD8GFP and NP1017-GAL4/+;UAS-mCD8GFP/+ are lateral views. Gr66a-GAL4/UAS-mCD8GFP is an anterior view. (0.48 MB PDF) [file pbio.1000147.s005.pdf]

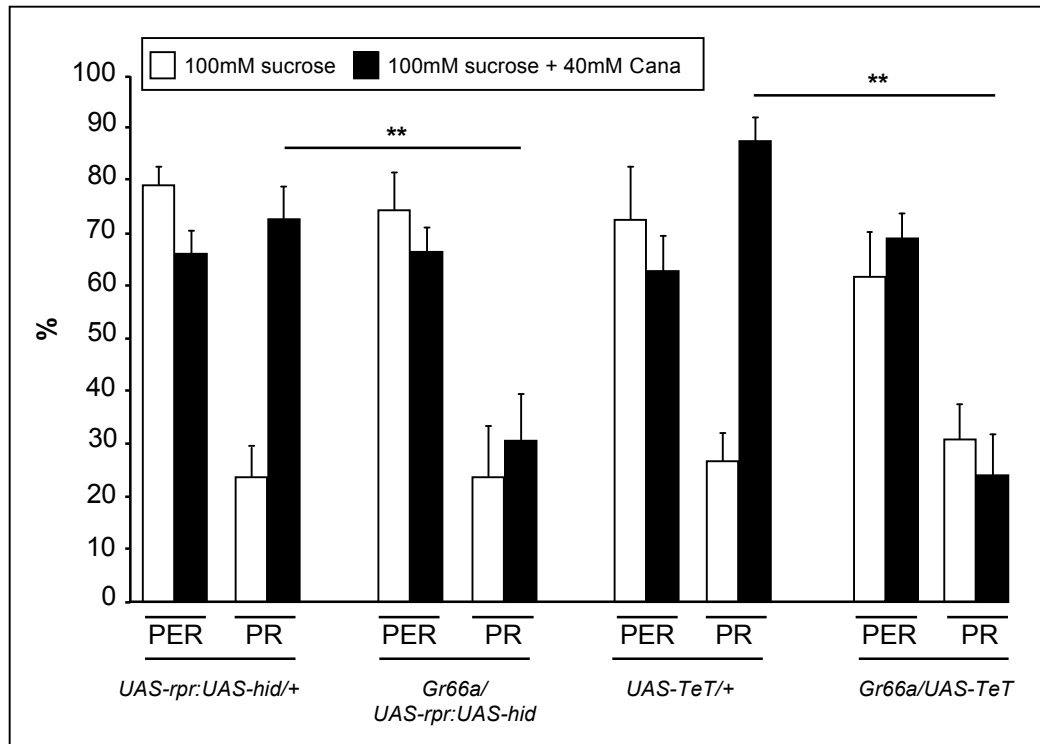

Figure S6

Supplement: Figure S6 — Gr66a-GRNs are necessary for L-canavanine-induced PR. By using the Gr66a-GAL4 driver, we targeted the expression of the proapoptotic genes (rpr and hid) and the tetanus toxin light chain (TeT) to kill and inactivate Gr66a-GRNs, respectively. Results show the percentage of PER and PR on controls (UAS-rpr:UAS-hid/+ and UAS-TeT/+), ablated (Gr66a/UAS-rpr:UAS-hid), and silenced (Gr66a/UAS-TeT) Gr66a-GRNs. Note that the PER was not affected in controls as well as when Gr66a-GRNs were ablated or silenced. Compared to controls, which show a high percentage of PR in presence of l-canavanine, the absence or the inactivation of Gr66a-GRNs abolishes the PR response. Behavioral analyses were performed as described in Figure 6. Error bars indicate SEM. Double asterisks indicate significant differences by t-test (p < 0.001). (0.06 MB PDF) [file pbio.1000147.s006.pdf]
