# Supplementary material for: Plant Insecticide L-Canavanine Repels Drosophila via the Insect Orphan GPCR DmX
Source: PLoS Biol. 2009 Jun 30;7(6):e1000147. doi: 10.1371/journal.pbio.1000147 (PMC2695807; doi:10.1371/journal.pbio.1000147)
Supplement: Figure S4 — The PER is not affected in rescue and loss of function experiments. (A) Knockdown of mtt expression by RNAi did not affect the PER response. Histograms show the percentage of PER of controls (NP4288/+, mttf06268/+;UAS-mtt RNAi/+, UAS-mtt RNAi/+, and Gr66a:mttf06268/+) and mtt heterozygous flies expressing the mtt RNAi construct either in NP4288-positive GRNs (NP4288/mttf06268;UAS-mtt RNAi/+) or in Gr66a-GRNs (Gr66a:mttf06268;UAS-mtt RNAi/+). Compared to controls, the down-regulation of mtt in NP4288-GRNs or in Gr66a-GRNs did not affect the PER response. Behavioral analyses were performed as described in Figure 6. Error bars indicate SEM. (B) Expression of DmXR in Gr66a, Gr5a, and NP1017 GRNs of mtt mutant flies did not affect the PER response. Histograms show the percentage of PER of controls (mttf06268/+;UAS-mtt/+), mtt mutant flies carrying one copy of each GRN GAL4 (Gr66a:mttf06268/mttf06268, Gr5a:mttf06268/mttf06268, and NP1017/+;mttf06268/mttf06268) and mtt mutant flies expressing mtt in bitter-, sugar-, and water-sensitive GRNs (Gr66a:mttf06268/mttf06268;UAS-mtt/+; Gr5a:mttf06268/mttf06268;UAS-mtt/+, and NP1017/+;mttf06268/mttf06268;UAS-mtt/+, respectively). For all genotypes, l-canavanine did not significantly affect the percentage of PER. Behavioral analyses were performed as described in Figure 6. (0.05 MB PDF) [file pbio.1000147.s004.pdf]

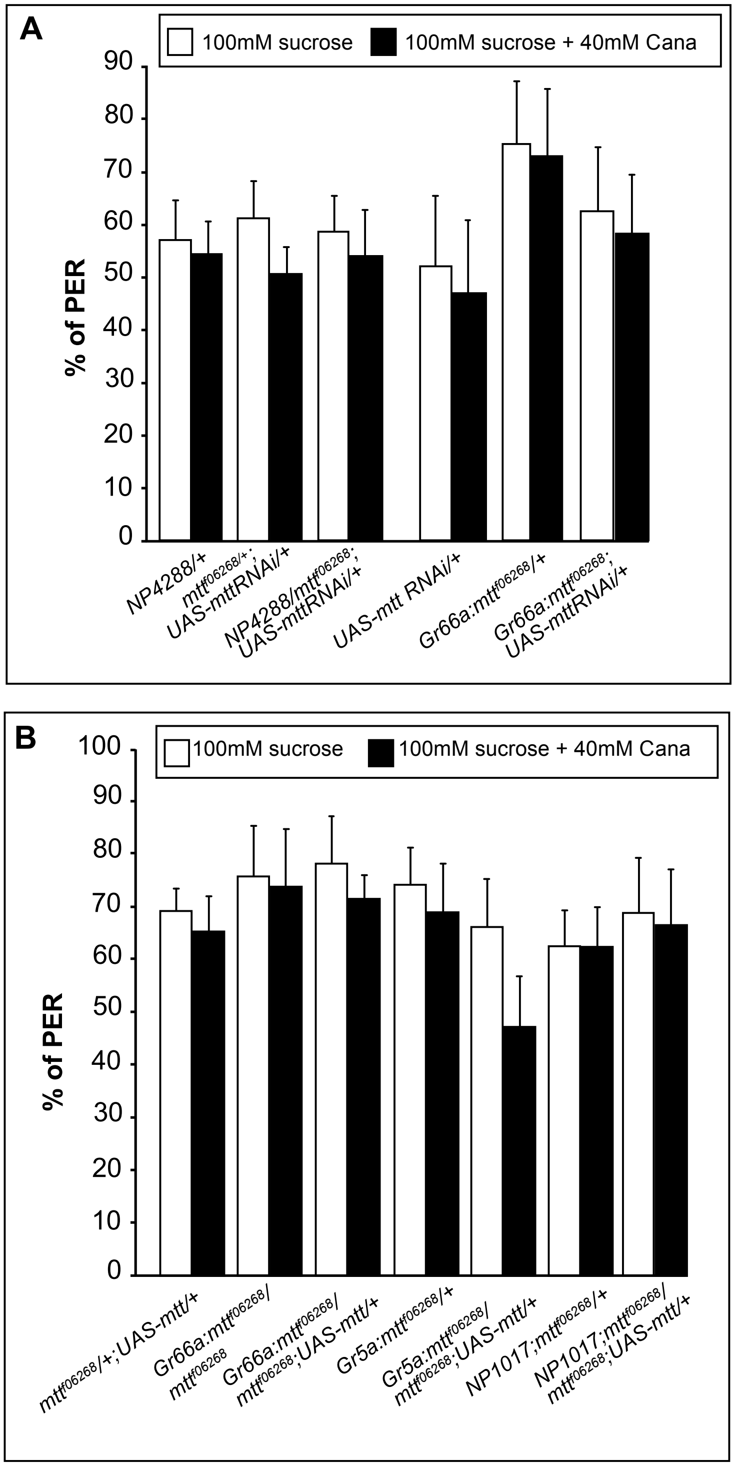
A
100mM sucrose
100mM sucrose + 40mM Cana
% of PER
0
10
20
30
40
50
60
70
80
90
NP4288/+
$mtt^{f06268/+}$;
UAS-mttRNAi/+
NP4288/mtt^{f06268};
UAS-mttRNAi/+
UAS-mtt RNAi/+
Gr66a:mtt^{f06268}/+
Gr66a:mtt^{f06268};
UAS-mttRNAi/+
B
100mM sucrose
100mM sucrose + 40mM Cana
% of PER
0
10
20
30
40
50
60
70
80
90
100
mtt^{f06268}/+;UAS-mtt/+
Gr66a:mtt^{f06268}/
mtt^{f06268}
Gr66a:mtt^{f06268}/
mtt^{f06268};UAS-mtt/+
Gr5a:mtt^{f06268}/+
Gr5a:mtt^{f06268}/
mtt^{f06268};UAS-mtt/+
NP1017;mtt^{f06268}/+
NP1017;mtt^{f06268}/
mtt^{f06268};UAS-mtt/+

Figure S4
